# An integrative review on physical restraint in adult critical care unit

**DOI:** 10.12688/f1000research.127358.1

**Published:** 2023-01-31

**Authors:** Janisha Kavumpurath, Kulanthayan KC Mani, Fatma Refaat, Navin Devaraj, Aneesa Abdul Rashid, Noor Airini Ibrahim

**Affiliations:** 1 Department of Nursing, University of Sharjah, Sharjah, United Arab Emirates; 2 Department of Nursing, University Putra Malaysia, Selangor, Malaysia; 3 Department of Community Health, Universiti Putra Malaysia, Selangor, Malaysia; 4 Department of Family Medicine, Universiti Putra Malaysia, Selangor, Malaysia; 5 Faculty of Medicine and Health Sciences, Universiti Putra Malaysia, Selangor, Malaysia

**Keywords:** physical restraint, critical care, mechanical ventilation, immobilization

## Abstract

**Background:** Physical restraints (PRs) are frequently used in adult critical care units to protect staff and prevent self-harm, despite the fact that they represent significant safety risks. Restraint complications may have an impact on the patient’s long- and short-term outcomes. This study aims to examine the recent evidence on the use of PR in adult critical care.

**Method:** This integrative review included the studies published between January 2009 and December 2019 and the literature search was conducted in July 2020. The databases searched included EBSCOhost, Ovid, ProQuest, PubMed, Wiley Online Library, SCOPUS, and ScienceDirect. The keywords included in the search were restraint, critical care, intensive care, ICU, mechanical ventilation, intubation, nursing, and experience. A checklist based on the CASP checklist and the JBI Critical Appraisal Tool was used to assess the methodological quality.

**Results:** The findings were evaluated and summarized into seven key topics after twenty-one publications were found to be evaluated. i) High prevalence of PR application in adult critical care unit; ii) determinants of PR applications; iii) types of PR in adult critical care units; iv) decision maker of PR; v) moral and ethical dilemma in PR application; vi) awareness and guidelines for PR applications; vii) common complications and use of sedation, analgesics, antipsychotic drugs in PR application.

**Conclusion:** The number of days PR is used is related to the risk of an adverse event. In order to standardize nursing practice, ICU nurses require greater training on the ideas of PR use. Evidence-based recommendations will assist critical care nurses in making the best judgments possible concerning the use of PR.

## Introduction

Delirium and increased anxiety are common issues in ICU patients, especially those who are on mechanical ventilation. Disturbing actions that lead to the endotracheal tube dislodging or interfering with other medical devices place these people at a higher risk of self-harm and even death. ^1^^,^^2^ Critically ill patients who are receiving intensive care are under a great deal of stress. A number of factors can contribute to the severity of the underlying disease, including the use of medical equipment, pain, and anxiety. Maintaining the highest degree of patient comfort and safety is crucial to the patient’s care in these situations. ^3^ Chemical and physical restraints (PRs) are frequently used in the intensive care unit (ICU) when caring for critically ill patients to reduce patient discomfort and anxiety. However, PRs result in considerable safety risks. ^3^^,^^4^

The use of any mechanical device or physical materials, or equipment that is attached to or around a patient’s body and that person cannot easily remove, restricting the patient’s range of motion or regular access to his or her body, is referred to as a PR. ^5^ It’s uncertain whether the usage of restraints is based on scientific facts or on custom and culture. ^6^

The patient who is physically restrained faces various risks, including lactic acidosis, incontinence, bone fracture, a sense of powerlessness and vulnerability, limited ROM, hospital-acquired pressure injury, venous thromboembolism, stress cardiomyopathy, respiratory depression, aspiration, choking, asphyxia, and even death. ^7^^,^^8^ The best ethical justification for PRs is that they prevent people from harming themselves. ^9^ PRs are often used despite the lack of evidence supporting their effectiveness. ^10^ PR practices have been studied in a number of countries. As a result, having a thorough understanding of current facts and practice is critical.

### Aim

With the use of the following questions, this review will look into the best available evidence on the use of PR in adult critical care in regard to: 1) prevalence of PR; 2) factors that influence PR; 3) the role the nurse plays in the implementation of PR; 4) the nurse’s experience caring for the patient with PR; and 5) the complications of applying PR.

### Search methods and strategy

The goal of this study is to conduct a thorough assessment of the available evidence on PR in the adult critical care unit. As a result, an integrative review was carried out. This integrative review includes quantitative, qualitative, mixed methodologies, and quality improvement projects from the accessible literature, referring to studies published between January 2009 and December 2019 and a literature search was conducted in July 2020.

EBSCOhost, Ovid, ProQuest, PubMed, Wiley Online Library, SCOPUS, and ScienceDirect were among the databases searched. Restraint, critical care, intensive care, ICU, mechanical ventilation, intubation, nurses, and experience were utilized as search terms. The “AND” and “OR” Boolean operators were employed.

**Figure 1.**
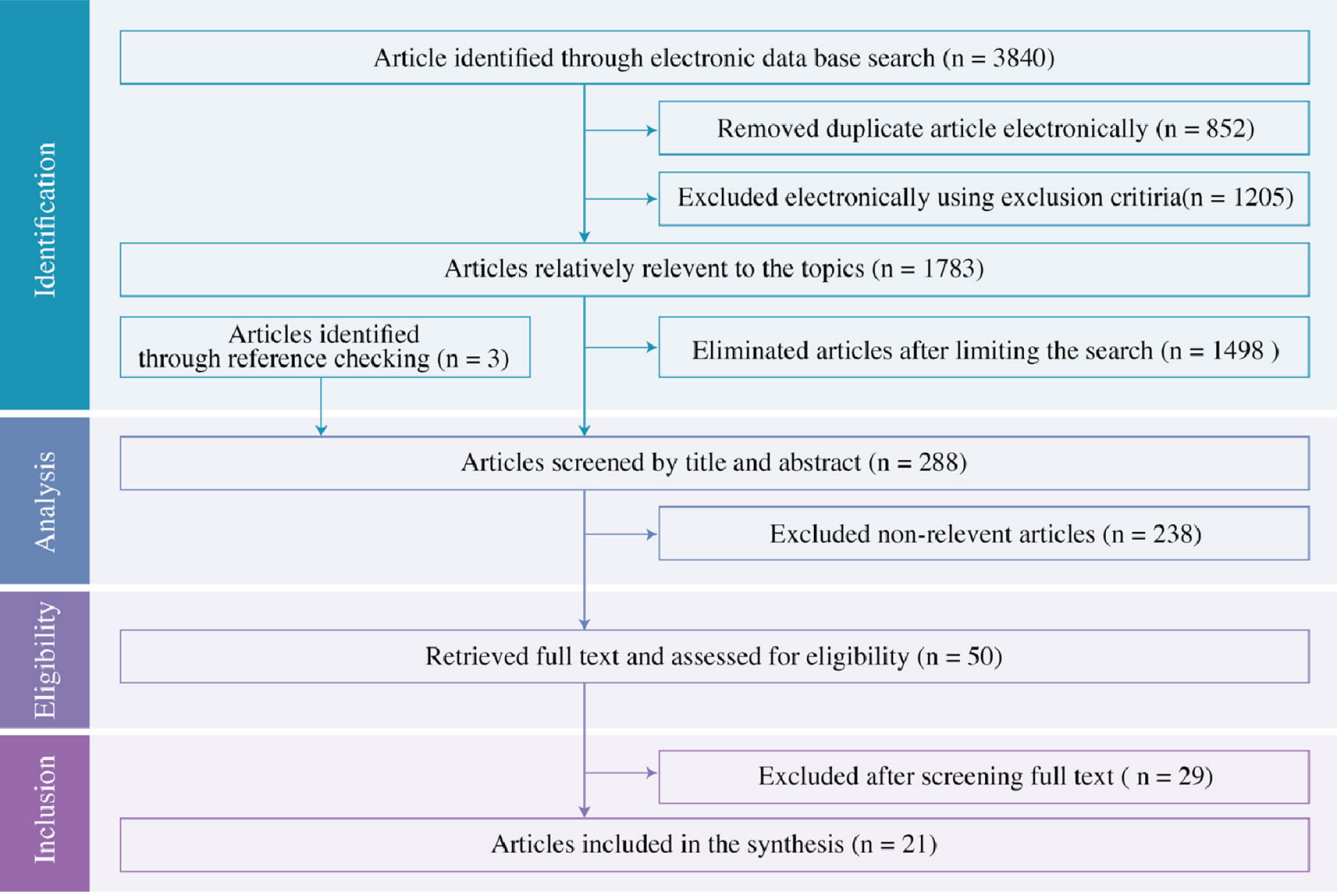
PRISMA flow diagram.

To map the search process, a flow diagram was used ( Figure 1). Initially, the database identified 3840 articles, and 852 duplicates were removed. From the remaining articles, 1205 were excluded electronically using exclusion criteria. Out of 1783, articles were found relatively relevant to the topic and 1498 articles were eliminated after limiting the search using the Boolean operator “AND”. A further seven were sourced from bibliographies and three of them were considered relevant to the screening. A total of 288 articles were considered for the title and abstract screening. 238 non-relevant articles were excluded and 50 full-text articles were retrieved. The screening process was done by two people independently. Twenty-nine of them were excluded after screening the full text and finally, 21 articles were considered for synthesis. EndNote X9.3.3 for Windows was used to manage the articles.

***Inclusion criteria***

Only peer-reviewed research involving patients or nurses in all types of adult ICUs was evaluated for this review, which was solely focused on the use of PR in adult critical care units. All full-text English papers with various techniques (quantitative research, qualitative studies, and quality improvement programs) are included.

***Exclusion criteria***

EndNote X9.3.3 electronically removed words in the title that contained animals ( *e.g.* rat), psychiatric, neonatal, and pediatric. Restraint and intensive care unit/critical care unit/ventilated/intubated/nurses/experience were used with the Boolean operator “AND” to narrow the search. Non-relevant papers were eliminated by manually scanning the title and abstract for exclusion criteria such as animal research, psychiatric/pediatric/neonatal units, and so on. The decision to include an article in the final stage was based on the study’s eligibility ^36^ and the content’s relevance. Review papers, editorials, conference abstracts, and commentaries were also excluded from this review.

***Eligibility of data***

The systematic review of literature for a particular intervention, condition, or issue is at the heart of evidence synthesis. The systematic review entails a number of sophisticated procedures that include an analysis of the existing literature (that is, evidence) and a judgment of the effectiveness or otherwise of practice. ^11^ The initial assessment of articles was done using the Critical Appraisal Sklls Programme (CASP) ^12^ checklist tool. This paradigm was chosen because it allowed for a systematic and rigorous approach to analyzing and evaluating article quality that could be used in both quantitative and qualitative methodologies. ^13^ The initial papers were screened using the inclusion/exclusion criteria. The literature was evaluated using the CASP tool’s three major questions: i) Is the research valid? (ii) What were the outcomes? (iii) Will the findings be usefully locally?. ^12^ The articles answered sufficiently for these three questions were further considered. According to Whittemore & Knafl, ^14^ two quality criterion instruments should be utilized during the review process to assure the authenticity, methodological quality, and informative value of the included research. Because of the wide sample frame, evaluating the quality of the research paper in integrative reviews might be difficult. The articles were re-evaluated using the more specific questions from the CASP ^12^ checklist tool and JBI (Joanna Briggs Institute) Critical Appraisal Tool. ^11^^,^^36^

The CASP tool and JBI Critical Appraisal Tool includes a set of detailed questions to enhance visualization of the data and to simplify this complex comparison of CASP tool and JBI Critical Appraisal Tool, a common checklist was adapted. Each article was assessed using the adapted checklist and used effectively to analyze the articles clearly and succinctly. While these checklists were designed to assess qualitative research papers, they were adjusted to include the essential criteria stated in both the CASP tool and the JBI Critical Appraisal Tool for a qualitative and quantitative approach. The modified checklist includes a clear aim, methodology, design, recruitment, data collection, researcher and participant relations, participants and their voices, ethical considerations, data analysis and rigor, findings, and research value.

***Data analysis***

By permitting the use of a combination of studies with diverse methodologies, the integrative review method is the only strategy that has the potential to play a larger role in nursing evidence-based practice. ^14^ Data analysis in research reviews necessitates the ordering, coding, categorization, and summarization of primary source data into a cohesive and integrated conclusion regarding the research problem. ^15^ Integrative evaluations draw on a variety of data sources to provide a more complete picture of the subject at hand. Combining many data sources, on the other hand, is difficult and time-consuming. ^14^ These challenges are approached by organizing data in a manageable framework. According to Whittemore & Knafl, ^14^ data reduction, data presentation, and data comparison are all part of the data analysis phase of integrative reviews. The phases of data reduction and presentation are extracting and coding data to be organized into a manageable framework, which will then be shown to increase the visualization of their linkages and patterns. The systematic categorization of data into patterns, themes, and variations is judged a suitable approach to this review framework due to the varied data from various approaches. And they proposed a consistent comparative approach for comparing integrative reviews, which is used in this review and given ( Table 2) along with main themes generated from the literature.

**Table 1.** Quality Assessment Checklist: Adapted from JBI and CASP. ^36^

Author, Year & Place of research conducted	Clear research questio ^1^/aim ^2^/objective ^1^	Methodology ^1,2^	Research design ^2^	Recruitment ^2^	Data collection ^1,2^	Researcher & participant relation ^2^/influence ^1^	Participants and their voices ^1^	Ethical consideration ^1,2^	Data analysis ^1,2^ and Rigours ^2^	Findings ^2^/interpretation of result ^1^	Research value ^2^/conlusion ^1^
Ahmadi *et al.*, 2019, Iran ^1^	✓	✓	✓	✓	✓	NA	NA	✓	✓	✓	✓
Gu *et al.*, 2019, China ^4^	✓	✓	✓	✓	✓	NA	NA	✓	✓	✓	✓
Jiang *et al.*, 2015, China ^16^	✓	✓	✓	✓	✓	x	✓	✓	✓	✓	✓
Hamilton *et al.*, 2017, Canada ^17^	✓	✓	✓	✓	✓	✓	✓	✓	✓	✓	✓
Kooi *et al.*, 2015, Netherland ^18^	✓	✓	✓	✓	✓	NA	NA	✓	✓	✓	✓
Luk *et al.*, 2014, Canada ^19^	✓	s	✓	✓	✓	NA	NA	✓	✓	✓	✓
Kandeel & Attia, 2013, Egypt ^20^	✓	✓	✓	✓	✓	NA	NA	✓	✓	✓	✓
Yönt *et al.*, 2014, Turkey ^21^	✓	✓	✓	✓	✓	NA	NA	✓	✓	✓	✓
Langley *et al.*, 2011, South Africa ^22^	✓	✓	✓	✓	✓	s	✓	✓	✓	✓	✓
Luk *et al.*, 2015, Canada ^23^	✓	✓	✓	✓	✓	NA	NA	✓	✓	✓	✓
Turgay *et al.*, 2009, Turkey ^24^	✓	✓	✓	✓	✓	NA	NA	✓	s	✓	✓
Benbenbishty *et al.*, 2010, Europe ^25^	✓	✓	✓	s	✓	NA	NA	✓	✓	✓	✓
Unoki *et al.*, 2019, Japan ^26^	✓	✓	✓	✓	✓	NA	NA	✓	✓	✓	✓
Ertuğrul & Özden, 2019, Turkey ^27^	✓	✓	✓	✓	✓	NA	NA	✓	✓	✓	✓
Guenette *et al.*, 2017, Canada ^28^	✓	✓	✓	✓	✓	NA	NA	✓	✓	✓	✓
Dolan & Looby, 2017, Massachusetts, US ^29^	✓	✓	✓	✓	✓	✓	✓	✓	✓	✓	✓
Hevener *et al.*, 2016, California, US ^30^	✓	✓	✓	✓	✓	s	x	✓	✓	✓	✓
Salehi *et al.*, 2019, Iran ^31^	✓	✓	✓	✓	✓	✓	✓	✓	✓	✓	✓
Balcı & Arslan, 2018, Turkey ^32^	✓	✓	✓	✓	✓	NA	NA	✓	✓	✓	✓
Mitchell *et al.*, 2018, Delaware, US ^33^	✓	✓	✓	✓	✓	NA	NA	✓	✓	✓	✓
Hall *et al.*, 2017, Virginia, US ^34^	✓	s	s	s	✓	NA	NA	✓	✓	✓	✓

✓ = Detailed coverage of screening question; s = Screening question covered; x = Screening question not covered; NA = Not applicable (Quantitative study).

1 = Joanna Briggs Institute (JBI) Critical Appraisal Tool; 2 = Critical Appraisal Skills Programme (CASP).

**Table 2.** Summary of Reviewed Articles and Key Findings.

Author, Year & Place of research conducted	Title & Objective	Research design & Sample	Findings
Ahmadi *et al.*, 2019, Iran ^1^	• Effect of Interventional Educational Programs on Intensive Care Nurses’ Perception, Knowledge, Attitude, and Practice about PRs. ○ Modify negative attitudes of intensive care nurses regarding the use of PRs.	▪ A quasi-experimental study with a pre-/post design with the census method ○ All nurses (n = 30) working in the ICUs had at least a bachelor’s degree and 1 year of working experience in ICUs.	▪ Interventional educational programs about the use of PR improved the level of knowledge and perception and led to a positive attitude and improved practice in nurses in the ICUs. ▪ All participants (100%) had used PRs before, and 20 of them (66.7%) had experienced complications caused by PRs.
Gu *et al.*, 2019, China ^4^	▪ Investigating influencing factors of PR use in China ICU: A prospective, cross-sectional, observational study. ○ Characterized the use of PRs in three ICU (ICUs) in a general hospital in Nantong, China. ○ Explored risk factors potentially related to PR use.	• Prospective, cross-sectional, observational study ○ 312 patients from three ICUs at a general hospital	▪ 61.2% of the patients were restrained. 56.5% of the patient’s bilateral upper limb restraints were used, and 22.3% of four-point restraints. ▪ The median length of PR use was 20 shifts (8hrs shift). 42.9% of patients were continuously restrained for more than 24 hours. In 75.9% of the cases, PR was used only once, and for the remaining, it was reapplied during the ICU stay. ▪ For 51.3% of patients, observational data was recorded in nurses' notes. There is no data for removal time, patient responses, or complications. Less than ^1^/ _3_ of patients gave informed consent for PR. ▪ Mostly restraint aimed to prevent the accidental removal of the medical devices to decrease any threat to the airway. ▪ Use of analgesics as an independent protective factor for PR use. The nurse’s record reflects a need for standard guidelines and policies for PR use in hospital ICUs in China. Age, delirium, mechanical ventilation, and analgesic use are risk factors related to PR use.
Jiang *et al.*, 2015, China ^16^	• Nurses’ perceptions and practice of PR in China. ○ Identify the perceptions and practice of PR in China.	▪ Mixed methods Descriptive study Qualitative interviews A quantitative cross-sectional survey ○ 18 nurses were interviewed. Of 330 nurses surveyed,	▪ Quantitative findings: PR is commonly used for patient safety. It is more often used in ICUs than in medical-surgical wards. more often used on the night shift. ▪ Qualitative findings: staff shortages and heavy workload were the most common reasons for PR. In-service/education is needed to minimize PR use in China.
Hamilton *et al.*, 2017, Canada ^17^	• The prevalence and incidence of restraint use in a Canadian adult ICU: A prospective cohort study. ○ Determine the extent of PR use in an urban teaching ICU. ○ To identify patient-specific factors that may contribute to the application of PRs. ○ Explore nurses’ rationale for use.	▪ Mixed-methods study. A prospective cohort design was used to collect patient data, and semi-structured interviews were used to collect nurse data. ○ In total, 30 permanent ICU staff nurses and 72 patients were admitted during the study period in the mixed medical-surgical ICU.	▪ Quantitative Data: Gender is not a contributing factor to the use of PRs. 75% of patients are restrained during their hospitalization. The prevalence rate was 358 restraint days per 1,000 ICU days. ▪ Four self-extubations were documented during the study (3 patients without restraint and one documented as a fear of being restrained). The presence of an endotracheal tube increased the odds of PR application eight-fold. Low nurse-to-patient ratios increased the odds of restraint use. All opioids (95%) or midazolam administration (95%) increased the odds of restraint use. ▪ Qualitative data: nurses’ interviews ▪ According to 57% of nurses, patients are restrained prior to interference with medical equipment. They did not consult with the physician before they restrained the patient. They had no alternative but to use PR to prevent extubation. ▪ Many of them expressed negative feelings about using PRs. Most nurses had empathy for tied patients and employed creative strategies to reduce their use or minimize the impact on the patient’s freedom. ▪ Deciding to remove restraints is dependent on patient behavior. ▪ The decision to remove the restraint was made when the patient demonstrated “cooperation with the nurse, was awake and aware of the surroundings,” and did not try to touch the tube, or when the patient was ready to have the endotracheal tube removed.
Kooi *et al.*, 2015, Netherland ^18^	• Use of PRs in Dutch ICU: A Prospective Multicenter Study. ○ Characterize the use of PR in ICU. ○ Prevalence, adherence to protocols, and correlates of the use of PR was determined. ○ For comparisons between ICUs, adjustments were made for differences in patients’ characteristics.	▪ A prospective, cross-sectional, observational multicenter study with two visits to each ICU ○ The sample included 379 patients and 346 nurses from 25 different Dutch ICUs.	▪ 23% were physically restrained during the researcher's visit. In 11 units (44%), the use of PR was more frequent, although this finding was not significant. 31% of nurses reported the use of a protocol for PR. ▪ The risk of PR is increased in patients with delirium or coma who cannot communicate verbally and are receiving psychoactive or sedative medications. There is no independent relationship between PR use and gender, illness severity, or nurse-to-patient ratio.
Luk *et al.*, 2014, Canada ^19^	• Predictors of PR use in Canadian ICUs. ○ Describe patterns of PR use in mechanically ventilated patients (prevalence, number of days of use, number of episodes of use). ○ Identify patient, treatment, and ICU/hospital characteristics associated with PR use and number of days of use.	▪ A secondary analysis of a prospective observational study ○ A sample of mechanically ventilated patients was admitted to 51 Canadian ICUs.	▪ PR was used with 374/711 patients. The average use of PR is 4.1 (SD 4.0) days (1 to 26 days). 83% applied only once, while the remaining reapplied multiple times during their ICU stay. ▪ Restrained patients experienced more adverse events and received higher daily doses of sedatives, analgesics, and antipsychotics. Use of sedative, analgesic, and antipsychotic drugs, agitation, heavy sedation, and the occurrence of an adverse event predicted PR use or the number of days used.
Kandeel & Attia, 2013, Egypt ^20^	• PRs practice in adult ICU in Egypt. ○ Investigate the practices of PRs among CCNs in El-Mansoura City, Egypt.	▪ A descriptive cross-sectional design with repeated observations ○ A convenience sample of two groups Group 1 consists of 275 physically restrained ICU patients. Group 2: 153 ICU nurses with at least one year of experience in one of the ICUs	▪ Restrained sites assessment is not documented in patients' records. Nurses’ self-report: PR is commonly (68%) used in ICUs. ▪ Individuals responsible for the decision to apply PR are nurses (58.2%), followed by physicians and nurses (41.1%). ▪ The most common PR behavior is an attempt to remove medical equipment (79.1%). 64.7% for resisting treatment or care and 46.4% for the attempt to get out of bed. Ensuring patient safety (96.1%) is the most important rationale for applying PR. ▪ Upper and lower limb restraints (37.9%) are the most common PR. Nurse-patient ratios and nurse workloads strongly contribute to the use of PR. ▪ Documentation of PR and assessment of restrained body parts is maintained by experienced nurses rather than novice nurses.
Yönt *et al.*, 2014, Turkey ^21^	• Examination of Ethical Dilemmas Experienced by Adult ICU Nurses in PR Practices. ○ Determine perceptions of ethical dilemmas by nurses in the application of PR.	▪ A descriptive and cross-sectional study ○ There are 55 intensive care nurses working in two hospitals' adult ICUs.	▪ 92.7% of nurses experienced the application of PR. 85.5% received training on ethics; 78.2% did not participate in training regarding ethical dilemmas. 94.5% of nurses reported that PR must be applied in ICUs. ▪ According to 70.9% of nurses, the decision of PR was jointly by the nurse and the physician and 25.5% by the physician alone. According to 65.5% of nurses, no family consent was taken for PR. 63.6% did not hesitate over PR. ▪ The reasons for PR are to prevent falls (25.4%), harming themselves (25.4%), removing the tubes (18.5%), maintaining the posture of the patient (3.8%), applying medical treatment (13.0%), and calm down the patient (13.9%). ▪ 36.4% of nurses felt difficulty in deciding PR use and experienced ethical dilemmas.
Langley *et al.*, 2011, South Africa ^22^	• Restraints in ICU-A mixed method study. ○ Provide a detailed description of the use of restraints in three ICU.	▪ Mixed methods—quantitative observational data and qualitative in-depth individual interviews ○ 5 medical practitioners and 15 registered nurses from 3 ICUs. There were 219 patients observed.	▪ Quantitative results: 1:1 was the nurse-patient ratio. 48.4% of the patients were restrained. 21.46% of patients were on analgesics and/or sedatives. 21.46% were restrained but not sedated. Patients were restrained for an average of 9 days (between 1 and 53). Ineffectively tied to nine patients ▪ Qualitative findings: restraint, a balancing act: all agreed that there is a place for the use of restraints in the ICU. Patients’ agitation needs to be assessed before PR. ▪ Proper PR use is described as a “balancing act” between its benefits and disadvantages. More than half of them stated that PR is primarily used to allow clinical staff to leave the patient unattended. ▪ Mostly, nurses are the decision-makers for PR. Not required Doctor’s prescription & they reluctant for that. Communication between the doctor, the patient, and their families is critical.
Luk *et al.*, 2015, Canada ^23^	• CCNs’ decisions regarding physical restraints in two Canadian ICUs: A prospective observational study. ○ Describe reasons for physical restraint application, alternative measures attempted, if any, prior to PR application, and reasons for restraint discontinuation.	▪ A prospective, observational study ○ Medical records of 141 patients from two medical-surgical ICUs	▪ Patients were mostly restrained using wrist restraints (91%). Four-point (all four limbs) restraints (.4%) and unilateral wrist-ankle and wrist-mitten restraint combinations are used infrequently (.2%). 83% of restraints were applied on the preceding night shift. ▪ Agitation 43%, restlessness 17%, and restraining as a precautionary measure to prevent accidents. In 33%, alternative measures were considered before PR. Device removal and maintaining patient safety 17% are the common reasons for PR. In 21% of patients, chemical restraint was used as an alternative. ▪ Restraint discontinuation was recorded for 57% patients, with the most common being a calm and cooperative patient for more than two hours.
Turgay *et al.*, 2009, Turkey ^24^	• PR Use in Turkish ICU. ○ Determine intensive care nurses’ reasons for the application and removal of PR. ○ Determine PR patterns used in Turkey ICUs.	▪ Descriptive and cross-sectional research designs. ○ 190 nurses work in the ICUs of 7 hospitals.	▪ There is no set policy or guidelines for restraint application practice. 84.7% PR without a physician’s order. 59.5% are not documented in nursing notes. Wrist and ankle ties are often (59.5%) used for PR. There was no difference in the frequency of restraint use between day and night shifts. ▪ 36.8% of complications were reported, and the most common one was skin breakdown. Maintenance of the placement of medical devices is the main (86.8%) reason for PR. ▪ Improved mental status is an important reason for the removal of restraints (68.9%).
Benbenbishty *et al.*, 2010, Europe ^25^	• PR use in ICU across Europe: The PRICE study. ○ Examine PR practices across European ICUs.	▪ Point prevalence survey conducted prospectively ○ In 9 countries, 34 general (adult) ICUs were observed and examined for charts or medical records by registered nurses and doctors. Out of 669 patients, physical and chemical restraints were used on 566 patients.	▪ The extent of PR varied across units. use of PR not related to the time of the week. PR is more likely in ventilated and sedated patients in the unit with a lower daytime nurse-patient ratio. ▪ Restlessness and delirium are the most common reasons for PR. Commercial wrist restraints are the most common. Patient safety is the dominant rationale for PR.
Unoki *et al.*, 2019, Japan ^26^	• PRs in ICU: a national questionnaire survey of PR use for critically ill patients undergoing invasive mechanical ventilation in Japan. ○ Describe the frequency of PR use among Japanese patients undergoing mechanical ventilation. ○ To verify the hypothesis that insufficient human resources have increased the frequency of PRs.	▪ Cross-sectional online open-anonymous survey ○ Nurses in the ICU with a patient-to-nurse ratio of no more than 2.	▪ PR is commonly used among mechanically ventilated patients in Japan. PR use varies among ICUs, irrespective of human resources and the proportion of beds in private rooms. ▪ A systematic approach is needed to reduce PR use in mechanically ventilated patients.
Ertuğrul & Özden, 2019, Turkey ^27^	• The effect of PR on neurovascular complications in ICU. ○ Investigate the effect of PR on the occurrence of neurovascular complications and their rate.	▪ A prospective observational cohort study ○ 90 patients from anaesthesia and the internal ICU.	▪ PR was used in the first 24 hours of hospitalization for 85.6% of patients.71.1% of patients had restraints on both wrists, with 17.8% having restraints on the right wrist and 11.1% having restraints on the left wrist. 71.6% was a roll of gauze, 17.6% was tough cuff material, and 10.8% was a green foam tie. ▪ After the first 24 hours, complications increased. A roll of gauze was used most commonly on the wrist. The roll of gauze and tough cuff material types led to an increase in redness. ▪ The duration of PR increases the risk of neurovascular complications. Nurses did not regularly check the restrained wrist and did not focus on the peripheral circulation.
Guenette *et al.*, 2017, Canada ^28^	• Psychotropic drug use in physically restrained, critically ill adults receiving mechanical ventilation. ○ Characterize psychotropic drug interventions before and after the use of PRs in critically ill adults receiving mechanical ventilation.	▪ A single-centre, prospective, observational study. ○ 93 patients who were admitted to the ICU	▪ All patients have 2-point Posey soft wrist restraints. Two patients started with the use of 4-point restraints and subsequently transitioned to 2-point restraints. ▪ The indication for PR was documented for 91 patients, and 2 patients were not documented. The most common documented indication was the prevention of treatment interference. ▪ The median duration of restraint was 21 hours (interquartile range, 9–70 hours); 43 patients (48%) were restrained for more than 24 hours. More patients received psychotropic drugs after PRs than before. ▪ Administration of opioids was more common after the use of PRs and accounted for more drug interventions. ▪ More patients received a psychotropic drug intervention after physical restraints were applied than before (86% vs. 56%). Administration of opioids was more common after the use of physical restraints (54% vs 20%) and accounted for more drug interventions (45% vs 29%). ▪ 16% remained over sedated and remained agitated before and after physical restraint. However, after the application of physical restraint, 20% of patients became over sedated and 6% less sedated. ▪ 10% of the patients become more agitated, compared to 8% less agitated.
Dolan & Looby, 2017, Massachusetts, US ^29^	• Determinants of nurses’ use of PRs in surgical ICU patients. ○ Describe nurses’ determinants of initiation and discontinuation of restraints in surgical ICU patients.	▪ Qualitative descriptive design. ○ 13 nurses working in the Surgical ICU.	▪ The initiation or discontinuation of PR is determined by the threat to patient safety (interruption or removal of monitoring and therapeutic devices). Surgical ICU experiences variable degrees of altered mental status. Restraints are used as an intervention to reduce self-extubation. Restraints are occasionally used to prevent injury to the staff because of patient behavior. ▪ Nurses identify patient-specific behaviors, including orientation and functional capacity, that determine restraint use. Nurses describe patient behaviors that predict the successful discontinuation of restraints without perceived negative effects. Thoughtful consideration is given to delirium patients due to the inconsistent responses to restraint use experienced by this patient population. ▪ The nurse’s ability to be vigilant in-patient care provides an atmosphere of security. Targeted nurse-driven interventions help in reducing restraint use, especially in delirious patients.
Hevener *et al.*, 2016, California, US ^30^	• Using a decision wheel to reduce use of restraints in a medical-surgical ICU. ○ Decrease use of restraints in a medical-surgical ICU. ○ To determine if a decision support tool is useful in helping bedside nurses determine whether or not to restrain a patient	▪ A pilot study with a quasi-experimental design was conducted. ○ Bedside nurses from the medical-surgical ICU	▪ In month one, 64 device dislodges occurred and 38% of the patients were restrained. During months 2 to 4, 51 devices were dislodged, and 12% of the patients were restrained. ▪ During the study, the use of restraints was reduced by 32%. There is a significant difference in restraint incidence before and after the use of the RDW, and there are no appreciable differences between the day shift and the night shift. For months 2 to 4, the RDW was used for 28.6%. ▪ 81% strongly agreed that the RDW was easily accessible. 62% slightly agreed that the RDW was useful in making decisions about the use of restraints. ▪ According to 85% of participants, the primary rationale for restraints is to prevent people from pulling out ET tubes. 77% felt comfortable removing restraints without a physician's order. ▪ 84% responded to using RDW on a regular basis. Many explained that an additional resource would also be beneficial and wanted to rely on their own clinical judgment on restraints.
Salehi *et al.*, 2019, Iran ^31^	• Factors behind ethical dilemmas regarding PR for CCNs. ○ Explore factors behind ethical dilemmas for CCNs overusing PR for patients.	▪ Qualitative study using conventional content analysis approach. ○ The 17 CCNs were purposefully recruited from the 4 ICUs.	▪ The outcome of using PR is the factor behind ethical dilemmas for CCNs. ▪ PR is used to ensure patient safety and prevent damage to patients and others. They were compelled to ignore patient rights and autonomy and use PR because of heavy workload and ward crowdedness. ▪ Other than PR, there is no choice but to use it to ensure patient safety. ▪ PR mentally damages patients and causes them problems such as fear of the ICU, depression, anger, aggression, restlessness, agitation, and anxiety. ▪ The risks of not using PR are delays in treatments, long hospital stays, falls, tissue trauma, pulling or removal of connections, heavy treatment costs, and even death. ▪ Nurses are not authorized to use PR without a medical order, but restraints are used without a physician’s order. Nurses perceive low support from physicians if a patient experiences a fall. ▪ Nurses felt conflicted due to the violation of patient rights and experience. Some of them felt uncertainty even after leaving the hospital.
Balcı & Arslan, 2018, Turkey ^32^	• Nurses' Information, Attitude and Practices towards use of PR in ICU. ○ Determine the knowledge, attitude and application levels of nurses working in critical care units about PR applied on patients.	▪ Descriptive and correlation studies ○ 158 nurses who work in medical-surgical intensive ICUs in hospitals and graduated from the faculty of health sciences	▪ Nurses deciding on the use of PR have higher scores than physicians. Nurses' attitude scores were found to be higher than those of physicians. Physicians' practice scores were found to be higher than those of nurses. ▪ Nurses have a sufficient level of information but negative attitudes and are insufficient in practice.
Mitchell *et al.*, 2018, Delaware, US ^33^	• Reducing Use of Restraints in ICU: A Quality Improvement Project. ○ Reduce and sustain the restraint rates to less than the national database means rates for all 5 ICU	▪ A quality improvement process Quantitative observational data from five ICUs with nine non-validated survey tools ○ 119 ICU nurses and ICU data from five ICUs	▪ The Restraint collaborative lowered the restraint rate. Bedside nurses engaged in evidence-based practice using the latest evidence-share willingly with colleagues. The restraint culture shifted from most patients to minimal use of restraints. ▪ The use of restraints was added to the daily-goals checklist and the need for restraints was assessed during daily inter-professional rounds. ▪ There is a lack of alternatives as a barrier to the removal of PRs. PRs may increase moral distress for nurses who care for these patients.
Hall *et al.*, 2017, Virginia, US ^34^	• Impact of a Restraint Management Bundle on Restraint Use in an ICU. ○ Explored the impact of a restraint management bundle on restraint use, quality, and safety outcomes.	▪ Secondary data analysis of the pre and post cohort groups ○ Data was extracted for 2701 patients from a 24-bed general ICU. There are 65 clinical RNs and 12 nursing care partners. Data from the EMR is used. In the pre-cohort group of 1339 patients and the post-cohort group of 1362 patients,	▪ There were 24.3% of patients restrained in the pre-cohort group compared to the post-cohort group (20.9%). The number of restrained patients per patient-day averaged 0.075 for the pre-cohort group compared with 0.059 for the post-cohort group. The number of restraint episodes per patient day averaged 0.191 for the pre-cohort group, compared with 0.133 for the post-cohort group. ▪ The average ICU length of stay was 3.64 days in the pre-cohort group compared with 3.60 days in the post-cohort group. ▪ The result shows a significant reduction in restraint use and duration, although ICU length of stay remained stable over time.

PR - Physical Restraint; ICU - Intensive care nurse; CCN - Critical care nurse; SD - Standard deviation; RDW - Restraint decision wheel.

## Results

Twenty-one articles were found to be suitable for this review ( Table 2). The studies were conducted in China, Japan, Turkey, Egypt, Iran, Canada, South Africa, the United States of America, the Netherland, and Europe. The quantitative data was gathered from various sources *via* observation and survey, while the qualitative data was gathered through face-to-face interviews. Each study has a different sample size.

The following are some of the themes that developed from the literature: i) high prevalence of PR application in adult critical care units; ii) determinants of PR applications; iii) types of PR in adult critical care units; iv) decision maker of PR; v) moral and ethical dilemma in PR application; vi) awareness and guidelines for PR applications; vii) common complications and use of sedation, analgesics, antipsychotic drugs in PR application.

### High prevalence of PR application in the adult critical care unit

The fact that PRs are most commonly employed in the adult critical care unit was obvious. Descriptive research in China found that ICUs used PRs more frequently than medical-surgical wards. ^16^ In this review, we found that 23% to 75% of the patients were restrained during their stay in the critical care unit. ^4^^,^^17^^–^^19^ Critical care nurses (CCNs) (68%) reported that PRs were most commonly used in ICUs. ^20^ According to 94.5% of CCNs physical restraint must be applied in ICU. ^21^ Most nurses working in critical care units experienced (68% to 100%) the use of PRs. ^1^^,^^21^ According to Langley *et al*., ^22^ all of the participants in their study agreed that PRs have a place in the ICU. And they stated that, before using PR the patient’s agitation must be assessed.

According to a study conducted in Canada by Luk *et al.,* ^23^ 83% of restraints were employed on the night shift and Jiang *et al.* ^16^ support that PR is more commonly used on the night shift. Turgay *et al.* ^24^ on the other hand, found no variations in the frequency of PR use between night and day shifts. PRs were utilized by 57% of nurses prior to the patient’s interference with medical equipment. ^17^

Five studies looked into nurse-to-patient ratios. According to Kandeel & Attia, ^20^ nurse workloads play a significant effect on the use of PR. Benbenbishty *et al.* ^25^ found that PRs are frequently utilized for sedated and mechanically ventilated patients in units with a lower daytime nurse-to-patient ratio. And according to Hamilton *et al.* ^17^ restraint use was more likely with low nurse-to-patient ratios. The nurse-to-patient ratio was mentioned in the other two studies, but there was no discussion of the relationship between the ratio and PR applications. ^18^^,^^22^ Regardless of personnel resources or the number of beds in private rooms, PR usage varied among ICUs in a cross-sectional online survey conducted in Japan. ^26^

According to Ertuğrul & Özden, ^27^ out of total restrained patients, 85.6% were initiated in the first 24 hours of their hospitalization. The duration of PRs varied from hours to days. An article from Canada explored the median duration of restraint as 21 hours ^28^ while a study from China found that patients stayed restrained for up to 20 shifts (8 hrs shift). ^4^ According to Langley *et al.* ^22^ and Luk *et al.* ^19^ the patients were restrained for an average of 9 and 4.1 days respectively. In a study, Hamilton *et al.* ^17^ found a prevalence rate of 358 restraint days per 1,000 ICU days. From two observational studies, out of total restrained patients, 42.9% to 48% were restrained for more than 24 hours. ^4^^,^^28^ Repeated application of PRs highlighted in some studies however, in most of the cases (75.9% - 83%) the PR was applied only once during ICU stay. ^4^^,^^19^

Documentation is vital when a nurse takes care of a patient. It will protect them from legal consequences arising from the outcome or lack of restraint. In a study, patients’ observational data were recorded in 51.3% of nursing notes and no data for removal time, patient responses, or complications were accurately recorded. ^4^ The indication for PR was reported for 91 patients and two individuals were not documented, according to Guenette *et al.* ^28^ More than 59% had no documentation of PR as per Turgay *et al.* ^24^ According to Kandeel & Attia, ^20^ patient records do not contain information about the assessment of restraint sites. According to them, experienced nurses maintained better documentation regarding PR application and assessment than novice nurses.

Many studies support the lack of documentation of PR applications among patients. ^4^^,^^20^^,^^24^^,^^28^ Hence, the prevalence rate of PR applications can be more than the available data suggests. The high prevalence rate of PRs shows its importance in the adult critical care unit, making it inevitable in the care of patients.

### Determinants of PR applications

The primary justification for restraints is patient safety. PRs are designed to prevent the unintentional removal of medical or therapeutic devices, hence reducing the threat to life. ^4^^,^^16^^,^^17^^,^^20^^,^^21^^,^^23^^,^^24^^,^^29^^–^^31^ The presence of an endotracheal tube raised the probabilities of PR application eight-fold, according to Hamilton *et al.* ^17^ who also emphasized the incidence of self-extubation in patients who were not restrained. Restraints were utilized as a preventative measure to prevent self-extubation. ^29^ The most prevalent documented indication in a observational study was the prevention of treatment interference. ^28^ Other indications for PR include prevention of falls or attempts to get out of bed, maintenance of posture & calming down the patient, and managing treatment resistance or care. ^20^^,^^21^

In this review, two studies show heavy workload and ward overflow with staff shortages as common reasons for PR. ^16^^,^^31^ According to the response of more than half of the samples in qualitative data collected by Langley *et al.,* ^22^ PRs were used primarily for the benefit of the clinical staff and to leave the patient unattended. Nurses face difficulties to identify an alternative as a barrier to the removal of PRs to ensure patient safety. ^17^^,^^30^^,^^31^ However, in a study, for 33% of patients, alternative measures were considered prior to PR, and in 21% of cases chemical restraints were used instead. ^23^

Restraints are sometimes employed to prevent injury to personnel or to oneself as a result of patient behavior such as agitation, delirium, restlessness, and varying degrees of impaired mental status. ^17^^,^^23^^,^^25^^,^^29^ Patients with delirium or coma, who are taking psychoactive or sedative drugs, and who are unable to speak vocally, are at a higher risk of receiving PR. ^18^ Establishing an alternative measure for reducing the use of PRs while ensuring patient safety remains a predominant challenge for nurses.

Patient’s requirements and desires must be addressed when applying for PR. Due to the variable responses to restraint treatment in this patient population, special consideration is given to delirium patients. The nurse’s capacity to maintain vigilance in patient care creates a safe environment. ^29^

The decision to release restraints is based on the patient’s behavior. Improved mental status (68.9%) is a vital indicator of the removal of restraint. ^24^ Restraints were removed from 57% patients who were generally calm and cooperative for more than two hours. ^23^ The nurses described that successful withdrawal of restraints without perceived negative effects is predicted according to patient behaviors. ^29^ According to the CCNs, the decision to remove the restraint was made when the patient cooperated with the nurse, was awake and aware of their surroundings, and did not attempt to touch the tube, or when the patient was ready to have the endotracheal tube removed. ^17^ Before stopping PR, however, it should be determined if there is a risk to the patient’s safety. Falls, tissue injuries, pulling or removing connections, delays in therapies, extended hospital stays, high treatment costs, and even death are all risks of not utilizing PR. ^31^

### Types of PR in adult critical care units

In critical care units, bilateral upper limb restraints were most commonly used. ^27^^,^^28^ ICU patients are also restrained using four-point restraints. ^4^^,^^20^^,^^24^ Among 141 patients in Canada, most were restrained using wrist restraints (91%). All-four-limb restraints were used on a few patients (4%) and combinations of unilateral wrist-ankle and wrist-mitten restraints were rarely employed (2%). ^23^

Benbenbishty *et al.* ^25^ explored the common use of commercial wrist restraints. In a study in Turkey, PR was used with tough cuff material, green foam tie, and a roll of gauze at a rate of 17.6%, 10.8%, and 71%, respectively. The roll of gauze was used most commonly on the wrist. ^27^ Hence, it can be articulated that the type of PR depends on the need of the patient and the material available in the unit.

### Decision maker of PR

The majority of the research in this review supports the nurse’s role as the principal decision-maker in the application and removal of PRs. In Egypt, nurses were responsible for 58.2% of the choice to use PR, while 41.1% was decided by both physicians and nurses. ^20^ According to Turgay *et al.,* ^24^ 84.7% of PR were applied without a physician’s order in Turkey and according to Balcı & Arslan, ^32^ nurses deciding on the use of PR have higher scores than physicians. However, Yönt *et al.* ^21^ stated that 70.9% were decided jointly by the nurse and the physician and 25.5% by the physician alone. Before restricting a patient in Canada, nurses did not confer with the physician. ^17^ Nurses are the primary decision-makers in PR, according to Langley *et al.* ^22^ from South Africa. Nurses in Iran are not allowed to use PR without a medical order; however, restraints are used without a physician’s order since nurses believe physicians are unsupportive if further complications arise from not using PR ( *e.g.* if a patient falls). ^31^ In California, 77% of nurses felt confident releasing restraints without a doctor’s order. Nurses wanted to rely on their own clinical judgment when determining whether or not to restrain a patient. ^30^

The literature shows that nurses play a significant involvement in the decision to use restraints on patients. In this review, no studies mention the role of patients or their families. Inconsistencies in the role should be addressed with a standard guideline that specifies each individual’s role (physician, nurse, patient, or family), and it should be very clear about a physician’s written order for PRs.

### Moral and ethical dilemma in PR application

Moral distress may increase among the nurses who care for patients under PR. ^33^ Many nurses voiced dissatisfaction with the use of PRs. Most of them empathized with restrained patients and established innovative ways to diminish PR use or lessen the impact on the patient’s freedom. ^17^ The ethical issues for CCNs are caused by the outcomes of utilizing PR. Because of the overwhelming workload and nursing unit overflow, they felt obligated to disregard patient rights and autonomy in favor of PR. Due to the breach of patient rights and experience, nurses were conflicted. Even after leaving their shift, some of them felt uncertain. ^31^ Nurses encounter ethical issues due to harm and benefit principles, according to Yönt *et al*. ^21^ In their study, according to 65.5 % of nurses, no family consent was obtained for PR, and 63.6 % of nurses have no reservations about PR.

In a Chinese study, only about a third of restrained patients gave informed consent for PR. ^4^ Communication between physicians, patients, and families is paramount. The proper application of PR should be a balancing act between its benefits and drawbacks. ^22^ These studies show the moral and ethical dilemmas facing nurses in the application of PR. Appropriate organizational policies will help nurses to overcome moral and ethical dilemmas. In addition, adequate training and education may be needed to prevent the psychological impact on the nurses.

### Awareness and guidelines for PR applications

A quality improvement project conducted in the US ^33^ shows a reduction in restraint rate after restraint collaboration and the restraint culture shifted from heavy to minimal use. The use of restraints was included in the daily-goals checklist, and the need for restraint was assessed daily during the multidisciplinary rounds. Furthermore the researcher added that, nurses who engage in evidence-based practice use the most up-to-date evidence and gladly share it with their colleagues. The impact of a restraint management bundle in an ICU on restraint utilization was studied by Hall *et al.* ^34^ When compared to the pre-cohort group, there was a reduction in the total number of restraint patients in the ICU in the post-cohort group (24.3% vs 20.9%). The average number of restrained patients per patient day decreased among the group (0.075 vs 0.059), and the average of restraint events per patient day also decreased (0.191 vs 0.133). According to the data, restraint use, and duration were reduced dramatically. The length of stay in the ICU, on the other hand, remained consistent over time (3.64 vs 3.6 days).

Targeted nurse-led interventions can help reduce the usage of restraints, especially in delirious patients. ^29^ Hevener *et al.* ^30^ conducted a quasi-experimental study in an ICU with the implementation of a restraint decision wheel (RDW) to reduce the usage of restraints, and the results demonstrate that restraints are gradually reduced in critical care units. In the first month, 64 devices were dislodged, and 38% of the patients were restrained; in the second to fourth months, only 51 devices were dislodged and 13% were restrained. During the research, the restraint used decreased to 32%. This study suggested 81% of nurses strongly agreed that the RDW was easy to use, and 62% thought that it was helpful in deciding whether or not to employ restraints, and 84% of nurses supported using the RDW on a regular basis. Many of them, however, stated that having another resource would be good.

In a descriptive and correlational study regarding PR, physician’s practice scores were found to be higher than those of nurses. Nurses have a sufficient level of information regarding restraint but have negative attitudes towards it. However, nurses’ attitude scores were found to be higher than those of physicians. And in practice, nurses’ skillset is lacking/insufficient. ^32^ This opened a floor for the training need of nurses about PRs. Ahmadi *et al.* ^1^ conducted Interventional Educational Program for ICU nurses to modify negative attitudes regarding the use of PRs. It leads to a positive attitude and improved the level of knowledge, perception, and practice among them.

In the descriptive and cross-sectional study, Yönt *et al.* ^21^ concluded that 36.4% of nurses felt difficulty in deciding on PR use and ethical dilemmas. The majority of them (78.2%) did not participate in training regarding ethical dilemmas. They recommended providing training for the nurses regarding ethical principles related to PR and PR application. In addition, they suggested having established policies for the use of PR in the ICU.

In a prospective multicenter study at Dutch ICUs, only 31% of nurses reported the use of a protocol for PR. ^18^ In this review, two studies highlighted the need for a set policy or guideline for the restraint practice ^4^^,^^16^ and Unoki *et al.* ^26^ suggested in-service education and the establishment of a systematic approach to reducing PR use in mechanically ventilated patients. However, the effectiveness and possibility of de-escalation in critically ill patients were not tested in any of the studies in this review.

The overall analysis of the data in this theme shows the high need for training for nurses regarding PR application. The establishment of a protocol or guidance will be more beneficial in the management of a patient with restraint. Implementation of Restraint Management Bundle, restraint decision wheel, and Targeted nurse-driven interventions can improve restraint application in ICU.

### Common complications and use of sedation, analgesics, antipsychotic drugs in PR application

Studies in this review show the negative outcomes of PRs like neuromuscular complications, increasing the need for sedation, analgesics, and antipsychotics. Turgay *et al.* ^24^ reported 36.8% complications and the most common one was skin breakdown. In Iran, 66.7% of the nurse had experienced complications caused by PRs. ^1^ According to prospective observational cohort research, most issues increased after the first 24 hours. An increase in redness was generated by the roll of gauze and tough cuff material. The length of PR raises the risk of neurovascular problems. Nurses failed to pay attention to peripheral circulation and failed to check the restrained wrist on a frequent basis. ^27^ Observational data from South Africa shows that 21.46% of patients with analgesics and/or sedatives. There were patients managed with restraint (21.46%) and no sedation. ^22^

More patients obtained psychiatric medicines after PRs were applied, according to a single-center, prospective, observational study done in Canada. According to that study, following the adoption of PRs, opioid administration became more widespread, accounting for more pharmacological interventions. Prior to PR, fewer people were treated with psychiatric drugs than subsequently (56% vs 86%). The administration of opioids was more common after the use of PRs (20 % vs 54%) and accounted for more pharmacological interventions (29% vs 45%). Before and after PR, 16% of 50 patients remained oversedated and disturbed. However, 20% of individuals got overly sedated after receiving PR, whereas 6% became less sedated. Furthermore, 10% of patients become more agitated, compared to eight percent who become less agitated. ^28^ According to Hamilton *et al.,* ^17^ all opioid or midazolam administration (95 %) increased the likelihood of restraint use. Analgesic, sedative, and antipsychotic medication use, excessive sedation, agitation, and the incidence of an adverse event all predicted PR use or days utilized. ^19^ However, according to Gu *et al.,* ^4^ analgesic use can be an independent protective factor for PR use.

PRs have a psychological effect on patients, causing issues including fear of the ICU, depression, anger, aggression, restlessness, agitation, and anxiety. ^19^ The impact of PR on the outcome of the physical and psychological elements was disclosed in this overview of studies on this topic. Restraint complications may have an impact on the patient’s long-term and short-term outcomes. Those issues can be avoided by assessing the restraints on a frequent basis.

## Discussion and implications

The magnitude of restraint utilization in adult critical care units is shown through a study of the current literature. PR is a common procedure in ICUs, according to this review. The review’s goal was driven by five questions, which were addressed and discussed under seven themes. Restraint is a technique for preventing the unintentional removal of medical or therapeutic equipment in order to reduce the risk of death. ^31^ It is frequently used to prevent treatment interference. ^28^ PR was extensively used to maintain medical devices among intensive care patients, and the type of PR was easily constructed by nurses. ^24^ The most prevalent reason for restraint removal noted by nurses in a study was the recovery of mental status. ^1^ CCNs will continue to utilize restraints until other ways have been scientifically proven to be effective in ICUs. ^24^

Restraints may be used as a result of a heavy workload and a shortage of staff nurses. ^16^^,^^31^ A systematic approach is needed to reduce PR use among mechanically ventilated patients ^26^ and implementation of the ABCDEF bundle can help avoid the use of restraints, prevent delirium and improve the management of delirium. ^33^

Nurses encounter practical, legal, and ethical challenges when administering PR to patients. In-service training on the use of PRs and ethical principles should be given to nurses to ensure that they make the best option possible on ethical issues. Furthermore, it is suggested that an evidence-based institutional policy governing the use of PR in ICUs be established. Create awareness among ICU nurses about the detrimental effects of PR on patients. ^21^

The importance of nurses as decision-makers in PR is highlighted in this literature. To improve patient safety, critical-care nurses should undergo in-service training on the use of PRs. ^20^ To ensure a patient’s safety, the least restrictive technique with the least risk and the most benefit to the patient should be used. It is necessary to analyze and resolve any unmet care needs or concerns. Restraints should not be used for the prevention of falls or the management of patient behavior as a routine. Consider a medical condition or symptom that suggests the necessity for immediate protective intervention to avoid violent behavior or getting out of bed. Bring the patient closer to the nurse’s station so that he or she can closely observe. Adequate staffing is required to monitor the patients closely. Non-self-destructive behavior should be described to the patient in simple terms, and the patient should be able to articulate his or her understanding. People who are agitated as a result of pain or an adverse reaction to a current drug or medication require intervention instead of applying restraints. ^35^ Evidence-based guidelines and educational interventions should be developed to support restraining practices. ^17^ This review underlines the lack of research on patients’ experiences with restraint throughout their ICU admission. There are variations in the way restraint is prescribed and documented. Furthermore, a restraint protocol and guidelines have the ability to improve the critical care unit’s restraint culture and practice.

## Conclusion

The use of PRs in the adult critical care unit is important for the treatment of life-threatening behavior. The number of days of PR usage is linked to the occurrence of an adverse event. As a result, long-term physical restrictions on patients should be avoided, and patients who are physically restrained should have a neuromuscular, circulatory, and skin assessment. The necessity for clear norms and policies for PR use is reflected in this review. Evidence-based guidelines will assist and support CCNs in making the best judgments possible about the use of restraints. To standardize nursing practice linked to the use of restraints for the protection of patient rights, and handling of ethical, legal, and emotional difficulties associated with PR, intensive care nurses require further education on the principles of restraint application. Furthermore, more study is needed to determine appropriate restraint alternatives.

## Data Availability

No data is associated with this article. Figshare: Quality assessment checklist for publications to be considered for review - Adapted from JBI and CASP, https://doi.org/10.6084/m9.figshare.21378213.v4. ^36^ This project contains the following extended data:
-
Table 1. doc (Example of quality assessment checklist). Data are available under the terms of the Creative Commons Zero “No rights reserved” data waiver (CC0 1.0 Public domain dedication). Repository name: PRISMA checklist used for integrative review, https://doi.org/10.6084/m9.figshare.21780632.v1. ^37^ Data are available under the terms of the Creative Commons Zero “No rights reserved” data waiver (CC0 1.0 Public domain dedication).

## References

[1] Ahmadi M Bagheri-Saweh MI Nouri B : Effect of Interventional Educational Programs on Intensive Care Nurses’ Perception, Knowledge, Attitude, and Practice About Physical Restraints: A Pre-/Postclinical Trial. *Crit. Care Nurs. Q.* 2019 Jan/Mar;42(1):106–116. 10.1097/CNQ.0000000000000244 30507671

[2] Reade MC Eastwood GM Bellomo R : Effect of Dexmedetomidine Added to Standard Care on Ventilator-Free Time in Patients With Agitated Delirium: A Randomized Clinical Trial. *JAMA.* 2016 Apr 12;315(14):1460–1468. 10.1001/jama.2016.2707 26975647

[3] Hofsø K Coyer FM : Part 1. Chemical and physical restraints in the management of mechanically ventilated patients in the ICU: contributing factors. *Intensive Crit. Care Nurs.* 2007 Oct;23(5):249–255. 10.1016/j.iccn.2007.04.003 17512733

[4] Gu T Wang X Deng N : Investigating influencing factors of physical restraint use in China intensive care units: A prospective, cross-sectional, observational study. *Aust. Crit. Care.* 2019 May;32(3):193–198. 10.1016/j.aucc.2018.05.002 30001953

[5] Martin B : Restraint use in acute and critical care settings: changing practice. *AACN Clin. Issues.* 2002 May;13(2):294–306. 10.1097/00044067-200205000-00013 12011600

[6] Teece A Baker J Smith H : Identifying determinants for the application of physical or chemical restraint in the management of psychomotor agitation on the critical care unit. *J. Clin. Nurs.* 2020 Jan;29(1-2):5–19. 10.1111/jocn.15052 31495002

[7] Ziaei M Massoudifar A Rajabpour-Sanati A : Management of Violence and Aggression in Emergency Environment; a Narrative Review of 200 Related Articles. *Adv. J. Emerg. Med.* 2018 Nov 29;3(1):e7. 10.22114/AJEM.v0i0.117 31172118 PMC6548084

[8] Mantovani C Migon MN Alheira FV : Manejo de paciente agitado ou agressivo [Management of the violent or agitated patient]. *Braz. J. Psychiatry.* 2010 Oct;32 Suppl 2:S96–S103. Portuguese. 10.1590/s1516-44462010000600006 21140077

[9] Crutchfield P Gibb TS Redinger MJ : The Conditions for Ethical Application of Restraints. *Chest.* 2019 Mar;155(3):617–625. 10.1016/j.chest.2018.12.005 30578755

[10] Mehrok S Belsiyal CX Kamboj P : The use of physical restraints- knowledge and attitude of nurses of a tertiary care institute, Uttarakhand, India. *J. Educ. Health Promot.* 2020 Mar 31;9:77. 10.4103/jehp.jehp_451_19 32490012 PMC7255578

[11] *Critical appraisal tools.* JBI.[cited 2020Jul26]. Reference Source

[12] *CASP checklists - CASP - critical appraisal skills programme.* CASP.[cited 2020 Jul 26]. Reference Source

[13] Higginbottom GM Richter MS Mogale RS : Identification of nursing assessment models/tools validated in clinical practice for use with diverse ethno-cultural groups: an integrative review of the literature. *BMC Nurs.* 2011 Aug 3;10:16. 10.1186/1472-6955-10-16 21812960 PMC3175445

[14] Whittemore R Knafl K : The integrative review: updated methodology. *J. Adv. Nurs.* 2005 Dec;52(5):546–553. 10.1111/j.1365-2648.2005.03621.x 16268861

[15] Cooper HM : *Synthesizing research: A guide for literature reviews.* Thousand Oaks, CA: Sage;2006.

[16] Jiang H Li C Gu Y : Nurses’ perceptions and practice of physical restraint in China. *Nurs. Ethics.* 2015 Sep;22(6):652–660. 10.1177/0969733014557118 25488757

[17] Hamilton D Griesdale D Mion LC : The prevalence and incidence of restraint use in a Canadian adult intensive care unit: A prospective cohort study. *Can. J. Crit. Care Nurs.* 2017;28(3):25–33. Reference Source

[18] Kooi AW van der Peelen LM Raijmakers RJ : Use of physical restraints in Dutch intensive care units: a prospective multicenter study. *Am. J. Crit. Care.* 2015 Nov;24(6):488–495. 10.4037/ajcc2015348 26523006

[19] Luk E Sneyers B Rose L : Predictors of physical restraint use in Canadian intensive care units. *Crit. Care.* 2014 Mar 24;18(2):R46. 10.1186/cc13789 24661688 PMC4075126

[20] Kandeel NA Attia AK : Physical restraints practice in adult intensive care units in Egypt. *Nurs. Health Sci.* 2013 Mar;15(1):79–85. 10.1111/nhs.12000 23302019

[21] Yönt GH Korhan EA Dizer B : Examination of ethical dilemmas experienced by adult intensive care unit nurses in physical restraint practices. Holist. *Nurs. Pract.* 2014 Mar-Apr;28(2):85–90. 10.1097/HNP.0000000000000013 24503745

[22] Langley G Schmollgruber S Egan A : Restraints in intensive care units--a mixed method study. *Intensive Crit. Care Nurs.* 2011 Apr;27(2):67–75. 10.1016/j.iccn.2010.12.001 21295485

[23] Luk E Burry L Rezaie S : Critical care nurses’ decisions regarding physical restraints in two Canadian ICUs: A prospective observational study. *Can. J. Crit. Care Nurs.* 2015 Winter;26(4):16–22. 26837121

[24] Turgay AS Sari D Genc RE : Physical restraint use in Turkish intensive care units. *Clin. Nurse Spec.* 2009 Mar-Apr;23(2):68–72. 10.1097/NUR.0b013e318199125c 19225286

[25] Benbenbishty J Adam S Endacott R : Physical restraint use in intensive care units across Europe: the PRICE study. *Intensive Crit. Care Nurs.* 2010 Oct;26(5):241–245. 10.1016/j.iccn.2010.08.003 20837320

[26] Unoki T Sakuramoto H Ouchi A : Physical restraints in intensive care units: a national questionnaire survey of physical restraint use for critically ill patients undergoing invasive mechanical ventilation in Japan. *Acute Med. Surg.* 2018 Dec 6;6(1):68–72. 10.1002/ams2.380 30652000 PMC6328904

[27] Ertuğrul B Özden D : The effect of physical restraint on neurovascular complications in intensive care units. *Aust. Crit. Care.* 2020 Jan;33(1):30–38. 10.1016/j.aucc.2019.03.002 31079994

[28] Guenette M Burry L Cheung A : Psychotropic Drug Use in Physically Restrained, Critically Ill Adults Receiving Mechanical Ventilation. *Am. J. Crit. Care.* 2017 Sep;26(5):380–387. 10.4037/ajcc2017677 28864434

[29] Dolan J Dolan Looby SE : Determinants of Nurses’ Use of Physical Restraints in Surgical Intensive Care Unit Patients. *Am. J. Crit. Care.* 2017 Sep;26(5):373–379. 10.4037/ajcc2017244 28864433

[30] Hevener S Rickabaugh B Marsh T : Using a Decision Wheel to Reduce Use of Restraints in a Medical-Surgical Intensive Care Unit. *Am. J. Crit. Care.* 2016 Nov;25(6):479–486. 10.4037/ajcc2016929 27802948

[31] Salehi Z Najafi Ghezeljeh T Hajibabaee F : Factors behind ethical dilemmas regarding physical restraint for critical care nurses. *Nurs. Ethics.* 2020 Mar;27(2):598–608. 10.1177/0969733019858711 31319750

[32] Balci H Arslan S : Nurses’ Information, Attıtude and Practices towards Use of Physical Restraint in Intensive Care Units. *J. Caring Sci.* 2018 Jun 1;7(2):75–81. 10.15171/jcs.2018.012 29977877 PMC6029653

[33] Mitchell DA Panchisin T Seckel MA : Reducing Use of Restraints in Intensive Care Units: A Quality Improvement Project. *Crit. Care Nurse.* 2018 Aug;38(4):e8–e16. 10.4037/ccn2018211 30068727

[34] Hall DK Zimbro KS Maduro RS : Impact of a Restraint Management Bundle on Restraint Use in an Intensive Care Unit. *J. Nurs. Care Qual.* 2018 Apr/Jun;33(2):143–148. 10.1097/NCQ.0000000000000273 28658189

[35] State Operations Manual Appendix A-Survey Protocol, Regulations and Interpretive Guidelines for Hospitals Transmittals for Appendix A.Cms.gov. 2020. [cited 2021Dec18]. Reference Source

[36] Kavumpurath J Mani KKC Devaraj NK : Quality assessment checklist for publications to be considered for review - Adapted from JBI and CASP.[Dataset]. *figshare.* 2022. 10.6084/m9.figshare.21378213.v4

[37] Kavumpurath J Mani KKC Ahmed FR : Prisma checklist used for an integrative review.Dataset. *figshare.* 2022. 10.6084/m9.figshare.21780632.v1

