# Supplementary material for: An integrative review on physical restraint in adult critical care unit
Source: F1000Res. 2024 Jun 14;12:114. Originally published 2023 Jan 31. [Version 2] doi: 10.12688/f1000research.127358.2 (PMC11301127; doi:10.12688/f1000research.127358.2)
Supplement: Peer review history

# Reviewer report: Reviewer response for version 2

**DOI:** 10.5256/f1000research.167201.r309245

**Published:** 2024-08-05

**Authors:** David Smithard

**Affiliations:** 1 University of Greenwich, London, UK

Thank you for asking me to review this paper. Restraint with in Critical Care and in fact most clinical settings is common depending on how you define 'restraint'. In the review there is a large discussion around staffing that is not alluded to in the conclusion. The conclusion, nor the review has made any comment on the fact that tube dislodgement occurs in people who are physically restrained. 

 The discussion is very good at high lighting alternative interventions to manage delirium which would then reduce the need for the se of restraints. 

 Standardising nursing practice guidance will not change things unless a more fundamental approach is taken to ensure that the environment within critical care is appropriate, patient identification improves and staffing levels are tackled.

Are the rationale for, and objectives of, the Systematic Review clearly stated?

Yes

Is the statistical analysis and its interpretation appropriate?

Not applicable

If this is a Living Systematic Review, is the ‘living’ method appropriate and is the search schedule clearly defined and justified? (‘Living Systematic Review’ or a variation of this term should be included in the title.)

Not applicable

Are sufficient details of the methods and analysis provided to allow replication by others?

Yes

Are the conclusions drawn adequately supported by the results presented in the review?

No

Reviewer Expertise:

Dysphagia epidemiology and rehabilitation. We have published in this area previously

I confirm that I have read this submission and believe that I have an appropriate level of expertise to confirm that it is of an acceptable scientific standard, however I have significant reservations, as outlined above.

## References

[1] : Physical Restraint in the Critical Care Unit: A Narrative Review. *New Bioeth* .2022;28(1) : 10.1080/20502877.2021.2019979 68-82 10.1080/20502877.2021.2019979 35083967

[2] : A Principlist Justification of Physical Restraint in the Emergency Department. *New Bioeth* .2021;27(2) : 10.1080/20502877.2021.1903152 176-184 10.1080/20502877.2021.1903152 33818319

# Reviewer report: Reviewer response for version 1

**DOI:** 10.5256/f1000research.139858.r247584

**Published:** 2024-03-06

**Authors:** Ziad Alostaz

**Affiliations:** 1 Sally Horsfall Eaton School of Nursing, George Brown College, Toronto, Ontario, Canada

Thank you for the opportunity to review this manuscript. The aim of this integrative review was to examine recent evidence on the use of physical restraints (PR) in adult critical care. This is an important topic, and while I appreciate the work conducted, I wonder about the novelty of the study, i.e., what does this review add to the literature? Are the methods/findings of this study novel? How do the findings of this review differ, expand, or complement the findings of the following reviews: Perez D, et. al. 2019 (ref 1), Alostaz Z, et. al. 2022 (ref 2).

 The rationale for and objective of the review is stated, but I would recommend more consistency. For example, while the abstract states the aim is to "examine the recent evidence on the use of PR in adult critical care," the body of the manuscript states that "with the use of the following questions, this review will look into the best available evidence on the use of PR in adult critical care in regard to: 1) prevalence of PR; 2) factors that influence PR; 3) the role the nurse plays in the implementation of PR; 4) the nurse’s experience caring for the patient with PR; and 5) the complications of applying PR." Furthermore, under the search methods, it was stated that "the goal of this study is to conduct a thorough assessment of the available evidence on PR in the adult critical care unit."

 Please note that there were no questions reported to support the sentence "with the use of the following questions."

 More details are needed to allow the replication of this review. For example, it would be helpful to provide the full search strategy for one of the databases (e.g., Medline).

 In the section discussing exclusion criteria, it appears that researchers primarily outlined how they limited the search rather than explicit criteria for excluding studies from the review. To enhance the reproducibility of this review, I recommend explicitly stating the predetermined criteria for exclusion. For example, exclusion of chemical restraints studies, studies conducted in any other setting outside ICUs, previous literature reviews, editorials, and conference abstracts, etc.

 Finally, I would recommend a review of the grammar used in the article as there was a mix-up of tenses (present, past, future). For example, under the aim it says "this review will look into...". Past tense should be used here, and there are many instances throughout.

Are the rationale for, and objectives of, the Systematic Review clearly stated?

Partly

Is the statistical analysis and its interpretation appropriate?

Not applicable

Are sufficient details of the methods and analysis provided to allow replication by others?

Partly

Are the conclusions drawn adequately supported by the results presented in the review?

Yes

Reviewer Expertise:

Physical restraint use in ICUs.

I confirm that I have read this submission and believe that I have an appropriate level of expertise to confirm that it is of an acceptable scientific standard, however I have significant reservations, as outlined above.

## References

[1] : Physical restraints in intensive care-An integrative review. *Aust Crit Care* .2019;32(2) : 10.1016/j.aucc.2017.12.089 165-174 10.1016/j.aucc.2017.12.089 29559190

[2] : Implementation of nonpharmacologic physical restraint minimization interventions in the adult intensive care unit: A scoping review. *Intensive Crit Care Nurs* .2022;69: 10.1016/j.iccn.2021.103153 103153 10.1016/j.iccn.2021.103153 34920932

# Reviewer report: Reviewer response for version 1

**DOI:** 10.5256/f1000research.139858.r161973

**Published:** 2023-02-22

**Authors:** Pei Boon Ooi

**Affiliations:** 1 Department of Medical Sciences, School of Medical & Life Sciences, Sunway University, Subang Jaya, Malaysia

The review listed a few aspects of physical restraints, which is a common procedure, in adult critical care units. The authors performed an integrative review which included comprehensively all research designs -i.e., quantitative, qualitative, mixed methodologies, and quality improvement projects. Twenty-one published articles were scrutinized according to the PRISMA protocols and the findings were evaluated and summarized into seven key topics which are insightful for future researchers to consider when it comes to adult critical care. These themes, especially the high prevalence of PR application in the adult critical care unit and the determinants of PR applications will be useful for consideration when it comes to the use of PRs in the adult critical care units. Additionally, the other important but often times silent aspects such as the moral and ethical dilemma, awareness and guidelines for PR applications were also identified and discussed. Most importantly, this integrative review underlined the lack of research on patients’ experiences with restraint throughout their ICU admission.

Are the rationale for, and objectives of, the Systematic Review clearly stated?

Yes

Is the statistical analysis and its interpretation appropriate?

Yes

Are sufficient details of the methods and analysis provided to allow replication by others?

Yes

Are the conclusions drawn adequately supported by the results presented in the review?

Yes

Reviewer Expertise:

As I work as a counsellor, my area of expertise may not be directly involved with critical care which may serve as a limitation if this is a clinical study. However, I have published a few systematic review papers.

I confirm that I have read this submission and believe that I have an appropriate level of expertise to confirm that it is of an acceptable scientific standard.
